# A Paralleled Multi-Task Learning-Based Framework for Single-Lead ECG Fine-Grained Noise Localization, Denoising and Signal Quality Assessment

**DOI:** 10.3390/s25237152

**Published:** 2025-11-23

**Authors:** Yating Hu, Qing Liu, Zheng Zhou, Weize Xu, Hong Tang

**Affiliations:** 1 School of Biomedical Engineering, Dalian University of Technology, Dalian 116024, China; yatinghu@mail.dlut.edu.cn (Y.H.); zhouzheng@mail.dlut.edu.cn (Z.Z.); 2 Department of Cardiology, The First Affiliated Hospital of Dalian Medical University, Dalian 116011, China; liuqing@dmu.edu.cn; 3 Department of Heart Center, Children’s Hospital, Zhejiang University School of Medicine, National Clinical Research Center for Child Health, Hangzhou 310052, China; 4 Liaoning Key Lab of Integrated Circuit and Biomedical Electronic System, Dalian University of Technology, Dalian 116024, China

**Keywords:** multi-task learning, ECG quality assessment, denoising, transformer

## Abstract

Wearable ECG monitoring devices have become indispensable in personalized healthcare. However, dynamic signal acquisition during daily activities often introduces transient noise, which complicates signal classification and denoising, and may compromise diagnostic reliability. To address this challenge, this study proposes an ECG preprocessing framework based on multi-task learning, in which a fine-grained noise localization task is introduced to guide and assist both ECG signal quality assessment and denoising. Built upon a Transformer backbone and optimized with three task-specific loss functions, the proposed model leveraged weak supervision and pathological ECG data to learn robust noise-invariant representations. This design incorporates intra-class awareness, enabling the model to overcome various noise within the same quality category and to perform adaptive denoising beyond conventional inter-class-based approaches. Extensive experiments demonstrated state-of-the-art performance in both denoising and quality assessment, with weighted average F1-scores ranging from 95.72% to 98.49% and classification accuracy exceeding 95.68%. Moreover, under extremely severe noise conditions, the signal-to-noise ratio (SNR) is improved from −1.95 ± 3.83 dB to 12.20 ± 2.51 dB while preserving waveform fidelity. After pruning and quantization, the model could be effectively compressed, thereby enhancing its suitability for real-time deployment in edge computing scenarios. Overall, the proposed method not only preserved diagnostically important ECG waveforms and provided interpretable noise localization but also offers an efficient and clinically relevant solution for large-scale, real-time ECG monitoring.

## 1. Introduction

Wearable ECG devices represent a key trend in the future of personalized healthcare, owing to their capability for continuous and real-time cardiac monitoring [1,2]. For patients with cardiovascular diseases (CVDs), subtle or transient abnormalities may remain undetected during brief clinical examinations. Continuous ECG monitoring in daily life enables the identification of such irregularities outside hospital settings [3]. This enhanced detection capability is necessary for early diagnosis and timely intervention, thereby preventing severe cardiac events and improving overall cardiac management.

Long-term ECG monitoring typically spans several hours, generating vast amounts of data. The resulting millions of ECG recordings pose significant challenges to the capacity and efficiency of existing computer-aided diagnostic algorithms [4,5]. Moreover, in real-world scenarios, transient noise is often introduced by factors such as body motion and changes in posture [6]. It is still intractable to separate the valuable ECG waveform and reveal the potential information from abnormal interference. Therefore, ECG signal preprocessing is a vital step to eliminate short-term and transient noises, thereby achieving a higher signal-to-noise ratio (SNR) and preserving as many useful signal segments as possible.

Previous studies have explored various approaches for ECG signal quality assessment. Traditional methods relied on handcrafted features and threshold-based rules, which were limited in generalization [7,8]. More recent deep learning-based models achieved significant improvements by learning signal representations directly from raw ECG data [9,10,11]. Most of these approaches solely focused on classification accuracy and neglected the intrinsic relation between signal quality and denoising. To address the challenges of ECG signal quality assessment and denoising, numerous studies have explored various deep learning strategies to enhance network learning and feature representation through auxiliary tasks [12,13,14].

However, there still exist some unsolved issues. First, quality assessment datasets typically have a small amount of data or a limited number of people from whom signals are collected. This can cause models with large-scale parameters to overfit easily or fail to guarantee the generalization of the model. Second, there is a lack of ECG data under pathological conditions in available quality assessment datasets. This may lead algorithms to mistakenly filter out high-quality ECG signals collected during illness, resulting in failure to warn of diseases. Third, a critical challenge lies in the extremely short time scale of transient noises. This brevity creates ambiguity during the labeling process and presents a fundamental problem for model learning, as it becomes difficult to extract discriminative features that are both robust and precise.

To mitigate these problems, this study proposed an ECG preprocessing method based on a multi-task learning framework, where a fine-grained noise localization task was used to guide and assist both ECG signal quality classification and denoising tasks. The objective is to achieve end-to-end joint inference from raw ECG data. It identifies and effectively denoises intermediate-quality segments, thereby converting them into clean signals, while directly discarding unacceptable ones.

The key contribution of our methodology can be summarized as follows:

1. A novel multi-task learning framework that unifies signal quality assessment, denoising, and fine-grained noise localization within a single model. Provision of interpretable outputs, including noise level graphs, which enhance the model’s transparency and trustworthiness for clinical decision support.
2. The fine-grained noise localization task enhances classification and denoising by introducing intra-class awareness, enabling adaptive denoising and clinically relevant quality assessment beyond conventional inter-class-only approaches.
3. We demonstrated high practicality for resource-constrained environments by reducing the model through quantization and pruning, making real-time deployment on edge devices feasible.

## 2. Related Works

Researchers have attempted to deal with the denoising strategies and rank the ECG signal quality. PhysioNet/Computing in Cardiology Challenge 2011 (CinC2011) called for improving the quality of ECGs collected using mobile phones, and participants proposed different approaches based on various signal quality indices (SQI) and machine learning [7]. These SQIs encompassed both morphological features, such as the intervals and amplitudes of local waves, and informatics features, such as multiscale entropy and autocorrelation. Clifford et al. utilized seven SQIs (including kurtosis, skewness, energy proportion of different frequency bands, etc.) to characterize the quality of ECG signals, and fed these features into artificial neural networks or SVMs for classification [8]. Lars et al. proposed a two-step algorithm, which first rejected macroscopic errors and then quantified the noise on a continuous scale [15]. Udit et al. combined wavelet decomposition, noise reconstruction and temporal feature extraction to detect, localize and classify various ECG noises [16]. The bSQI was proposed to describe the degree of agreement or uniformity between the results produced by two independent QRS detectors. Liu et al. utilized up to ten QRS detectors to redefine bSQI from the combination of any two QRS detectors to test which combination outputs the highest performance [17]. Liu et al. developed a novel IoT-based wearable 12-lead ECG Smart Vest system for early detection of cardiovascular diseases [18]. A novel way for ECG quality assessment was proposed based on the posterior probability of an artifact detection classifier [19]. Zhang et al. compared the four algorithms on 27 linear and nonlinear features, including features derived from the encoding Lempel-Ziv complexity (ELZC) [20].

In contrast to the aforementioned machine learning techniques, deep learning approaches directly input ECG signals into convolutional neural networks (CNNs) with a large number of parameters for quality label learning [9,10,11,21], which significantly enhanced computing speed and algorithm performance. Researchers have attempted to address the quality assessment problem of clinical ECG and provided an effective 7-layer Long Short-Term Memory (LSTM) neural network [22]. To further explore the potential noise information embedded in signals, previous studies have employed methods such as continuous wavelet transform (CWT), wavelet scattering, and short-time Fourier transform (STFT) to convert one-dimensional ECG signals into two-dimensional time-frequency representations. These transformed signals were then combined with deep neural networks (DNN) for enhanced quality assessment [23,24,25,26]. It is evident that deep learning methods have been widely applied in the field of ECG quality assessment. However, due to the more complex acquisition and labeling processes of ECG signals, the lack of large-scale ECG datasets remains a significant challenge in research.

To further investigate the problem of ECG signal quality assessment and noise separation, as well as to enhance model performance, various research approaches have been dedicated to improving network learning capabilities from different perspectives. Multi-task learning was applied for the combination of denoising and signal quality assessment [12,13], even for noise localization [27]. Due to the lack of fine-grained labels, FGSQA-Net was proposed for fine-grained quality assessment for various ECG recordings and was suitable for ECG monitoring using wearable devices [28]. Moving beyond the inherent limitations of human-specified properties, a data-driven quality indicator was introduced, learned directly from data using an unsupervised autoencoder [29]. For detecting unknown anomalies, an unsupervised SQA method by modeling the SQA of ECG as a problem of anomaly detection, to correct the hypersphere learned solely by the high-quality ECG samples [30]. However, previous studies have largely neglected the challenge of transient noise inherent to dynamic ECG acquisition, which can severely distort waveform morphology and compromise downstream analysis.

## 3. Methodology

**Figure 1:**
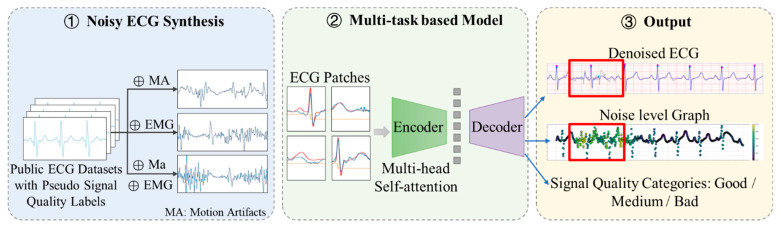
Overall framework of the proposed method.

The overall framework of the proposed methodology, as illustrated in Figure 1, comprises two core components:

1. Noisy ECG Synthesis: To enhance model robustness, clean ECG signals were dynamically combined with multiple noise sources (muscle artifacts and electrode motion noise) during training, generating a comprehensive dataset with weak-supervision noise level labels that simulated real-world noisy ECG recordings.
2. Multi-Task Learning Architecture: A unified neural network was optimized through three dedicated loss functions, each targeting distinct objectives: denoising fidelity, signal quality classification, and noise localization.

### 3.1. Noisy ECG Synthesis

Baseline wander (BW), electrode motion artifacts (EM), and muscle artifacts (MA) were the primary sources of interference in the process of dynamic ECG acquisition. ECG waveform usually has a frequency range of 0.05–45 Hz for clinical monitoring. Temporal and spectral characteristics of the mentioned ECG noise sources can be summarized as follows.

1. Baseline Wander: Baseline shifts and abrupt drifts occur due to factors such as respiration, physical activity. The frequency spectrum of baseline wandering typically spans from 0.05 to 1 Hz. Due to its low-frequency nature, it does not affect the morphology of the ECG waveform. Therefore, this paper does not take it into consideration, which also distinguishes this study from other studies.
2. Motion artifacts: Motion artifacts manifest as transient baseline alterations or rapid shifts, resulting from changes in electrode-skin impedance associated with electrode movement. The amplitude and duration of motion artifacts are notably over the peak-to-peak ECG amplitude and range from 300 to 500 ms [6]. Severe motion artifacts can distort the ST segment, which may lead to misdiagnoses of conditions such as myocardial infarction and other abnormalities associated with the ST segment [6].
3. Muscle Artifacts: also known as electromyogram (EMG) interference, arise from the electrical signals produced by muscles during contraction or sudden body movements. Typically, the intensity of EMG is about 10% of the ECG signal amplitude, and its frequency mainly spans 0.02 to 10 kHz, overlapping with the ECG frequency band in the range of 0.01 to 100 Hz. Consequently, eliminating muscle artifacts presents a considerable challenge, as it must be performed without distorting the clinical features that are crucial for diagnosing various ECG arrhythmias [31].

**Figure 2:**
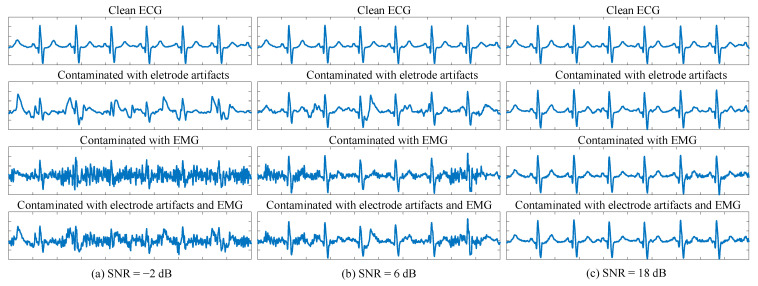
Representative examples of each quality category. (**a**): “Bad” with SNR of −2 dB. The first row shows the original clean ECG signal. The second and third row shows original signal mixed with electrode artifacts and EMG, respectively. The last row shows original signal mixed with both electrode artifacts and EMG. (**b**): “Medium” with SNR of 6 dB. (**c**): “Good” with SNR of 18 dB.

Considering the characteristics mentioned above, simulated ECG signals with different noise levels were generated from clean recordings according to the SNR rules in Table 1. Only EM and MA noises were considered in SNR computation, while BW was excluded due to its negligible impact on waveform morphology. The quality criteria are also summarized in Table 1, in accordance with [32]. The “Good” category was defined based on physicians’ experience, where noise did not hinder diagnostic interpretation. Representative examples of each quality level are shown in Figure 2.

### 3.2. Proposed Model Based on Multi-Head Self-Attention Mechanism

The proposed model is an end-to-end deep neural network based on a Seq2Seq architecture comprising an encoder and a decoder.

The encoder design was inspired by the Vision Transformer (ViT) [33], which introduced the concept of dividing images into fixed-size patches and processing them as “tokens” through a Transformer architecture—a milestone in patch-based deep learning. Building upon this idea, PatchTST [34] extended patch-based modeling to time series within a Seq2Seq framework, effectively leveraging Transformers for temporal representation learning.

**Figure 3:**
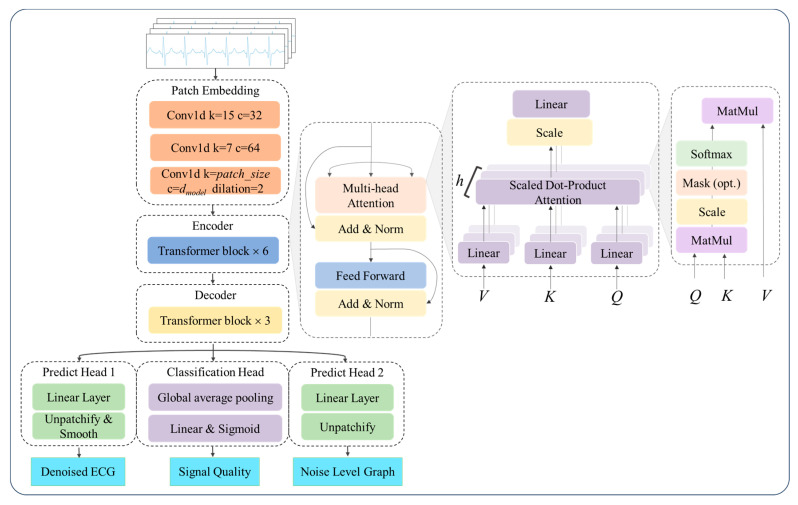
Architecture of the proposed model. Basic structure of a Transformer.

Following these insights, the input ECG signals were segmented into overlapping patches and then encoded using the multi-head self-attention mechanism (MHSA) based encoder [35] to derive the context vector representing the global temporal dependencies within the ECG sequence. Unlike ViT, a 1-D convolutional module was employed for patch embedding. Specifically, the first two convolutional layers used kernel sizes of 15 and 7 with a stride of 1, each followed by batch normalization and a ReLU activation function, while the final layer adopted a kernel size equal to the patch size and a stride of half the patch size. The encoder was composed of several identical blocks, each containing two core sub-layers: Multi-Head Self-Attention mechanism and Position-wise Feed-Forward Network (FFN) [35], shown in Figure 3. The computational steps can be summarized by the following formulas:

(1) $$Attention\left(Q,\;K,\;V\right)=softmax\left(QK^{T}/\sqrt{d_{k}}\right)V$$

(2) $$MultiHead\left(Q,\;K,\;V\right)=Concat\left({head}_{1,}{head}_{2,}\cdots ,{head}_{h}\right)W_{O},\;where\;{head}_{i}=Attention\left(QW_{i}^{Q},\;KW_{i}^{K},\;VW_{i}^{V}\right)$$

(3) $$FFN\left(x\right)=max\left(0,xW_{1}+b_{1}\right)W_{2}+b_{2}$$

where $Q\in R^{L\times d_{q}}$, $K\in R^{L\times d_{k}}$ and $V\in R^{L\times d_{v}}$ are the matrices of query, key, and value; *L* is the feature length; $d_{q},\;d_{k}$ and $d_{v}$ is the dimension of query, key and value; $W_{i}^{Q}\in R^{d_{model}\times d_{q}},\;W_{i}^{K}\in R^{d_{model}\times d_{k}}$, $W_{i}^{V}\in R^{d_{model}\times d_{v}}$ and $W_{i}^{O}\in R^{{hd}_{v}\times d_{model}}$ are the linear projection matrices.

The basic block of the decoder featured a structure similar to that of the encoder but with reduced complexity, enabling it to decode the encoded representation back into a meaningful output sequence. In the proposed model, positional encoding was omitted for two main reasons: first, ECG signal quality classification relied on local features of the signal, such as noise levels and waveform integrity, rather than on global temporal order; second, the input ECG signals were segmented into shorter patches, and explicit positional information was not required to capture temporal dependencies. Since data flowed through the network in the form of patches, an unpatchify operation was performed on the decoder output to obtain a vector of the same length as the original input signal. An additional 1-D convolutional layer was applied to the prediction head to smooth the predicted noise levels and denoised ECG signals. For the quality classification task, global average pooling was employed in the classification head to ensure that each patch contributed equally to the final prediction. Classification probabilities were generated via a fully connected layer with three output neurons. The architecture of the model is depicted in Figure 3, and the detailed configuration is listed in Table 2.

By using convolutional kernels of different sizes, the convolutional layers could capture multi-scale features in the ECG signals, such as short-term local fluctuations and long-term trend changes. The Transformer could integrate these multi-scale features to form a global, context-rich representation. The convolutional layers introduced locality bias, emphasizing that signals at adjacent time points are correlated, which aligned with the characteristics of ECG signals. The Transformer introduced global bias, capable of capturing relationships between distant time points, which was crucial for processing long sequences of ECG signals.

### 3.3. Loss Functions

The proposed multi-task framework jointly optimized three complementary objectives: denoising fidelity, signal quality classification, and noise localization.

#### 3.3.1. Denoising Loss

In terms of ECG denoising, the model functioned as a Denoising Auto-Encoder (DAE), utilizing a weighted reconstruction loss to optimize the trainable parameters. The customized loss function $L_{denoising}$ achieved an adaptive denoising optimization for labeled signals by integrating mean squared error (MSE) with wavelet decomposition-aware feature enhancement, which could be expressed as follows:

(4) $${\tilde{y}}_{i}=\left\{\begin{array}{c} 0,\;if\;y_{i}=“Bad”\\ 1,\;if\;y_{i}=“Good”\;or\;“Medium” \end{array}\right.$$

(5) $$L_{base}^{\left(i\right)}\left(t\right)={\left(x_{pred}^{\left(i\right)}\left(t\right)-x_{target}^{\left(i\right)}\left(t\right)\right)}^{2}$$

(6) $$L^{\left(i\right)}\left(t\right)=\left\{\begin{array}{c} \alpha \cdot L_{base}^{\left(i\right)}\left(t\right),\;\;ifa^{\left(i\right)}\left(t\right)>0\\ L_{base}^{\left(i\right)}\left(t\right),\;\;otherwise \end{array}\right.$$

(7) $$L_{denoising}=\frac{\sum_{i=1}^{N}{\tilde{y}}_{i}\left(\frac{1}{T}\sum_{t=1}^{T}L^{\left(i\right)}\left(t\right)\right)}{\sum_{i=1}^{N}{\tilde{y}}_{i}}$$

where $y_{i}$ represents the real quality category of sample *i* in each batch, *i* = 1, 2, 3,…, *N* and *N* is the batch size; ${\tilde{y}}_{i}\in \left\{0,1\right\}$ represents a normalized binary weight mask; $x_{pred}^{\left(i\right)}\left(t\right)$ and $x_{target}^{\left(i\right)}\left(t\right)$ are clean ECG and denoised ECG, respectively; $a^{\left(i\right)}\left(t\right)$ are wavelet coefficients; T is the length of the sample. For samples with the label “Good”, wavelet decomposition was performed on target signals to extract critical waveform components. The loss in these regions was amplified by the factor α, forcing the model to prioritize reconstructing key morphological features. Meanwhile, a weighted sum was computed using labels as masks (0 for “Bad”). By dynamically adjusting loss weights through time-frequency-domain feature perception, it preserved fundamental denoising capabilities while balancing global error minimization and local feature fidelity. It is particularly suited for ECG signal denoising tasks that require preserving specific waveform morphologies, ensuring robust recovery of diagnostically critical patterns. Designing a weighted loss based on the physiological importance of noise regions enhanced the penalty for errors in critical areas. By integrating dynamic weight adjustment, the model’s performance in key physiological regions of the ECG was further improved.

#### 3.3.2. Quality Classification Loss

Quality Classification loss $L_{cls}\;$ used cross-entropy loss, which is the preferred loss function for classification tasks, as it measures the difference between predicted probabilities and the true distribution, driving the model to optimize.

(8) $$L_{cls}=-\sum_{i=1}^{C}y_{i}log\left({\hat{y}}_{i}\right)$$

#### 3.3.3. Noise Level Estimation Loss

The Noise level Estimation loss $L_{level}$ was introduced to enable fine-grained noise localization. The prediction of noise probability at each sampling point in the ECG signal was formulated as a sequential regression problem, optimized using the mean squared error (MSE) loss. MSE is well suited for this task, as it directly measures the squared difference between predicted probabilities and the reference labels, thereby reflecting the accuracy of point-wise noise estimation.

**Figure 4:**
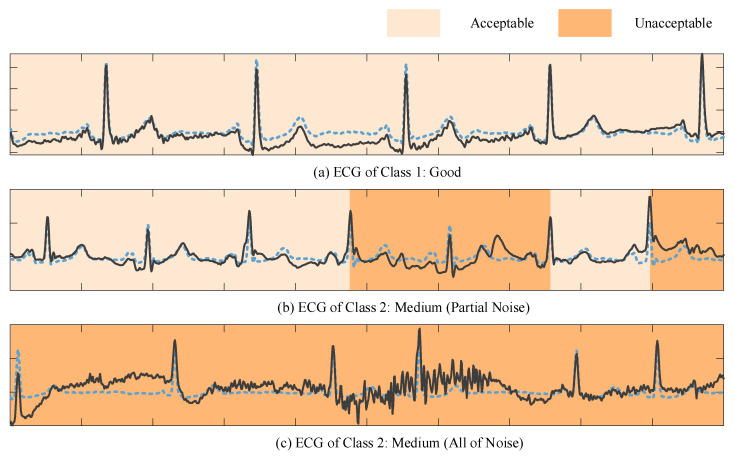
Noise level graph of synthetic ECG. (**a**) ECG of class 1. (**b**) ECG of class 2 with partial noise during some R-R intervals. (**c**) ECG of class 2 with noise during every R-R interval. The blue dotted lines represent original clean ECG signals. After the addition of noise interference, the noisy ECG signals were obtained, as indicated by the black lines in the figure.

Since dense and fine-grained noise annotations are prohibitively labor-intensive, we employed a weakly supervised strategy to infer noise probabilities computationally. Specifically, pseudo labels were derived by combining SNR and Pearson correlation coefficients within each R–R interval. These weak labels, inherently noisy due to their rule-based generation, assigned a probability of 1 to segments with indistinct fiducial points (unacceptable) and 0 to those with well-preserved fiducial points (acceptable). As illustrated in Figure 4, the resulting noise level graph highlights regions of varying noise intensity, where darker colors indicate a higher likelihood of unacceptable noise.

Beyond providing coarse noise supervision, this mechanism also introduces an element of intra-class awareness. ECGs belonging to the same quality category (e.g., “medium quality”) often exhibit heterogeneous noise patterns, such as localized EMG interference versus transient electrode motion artifacts. By learning from weak noise labels at the segment level, the model became sensitive to these intra-class variations, enabling adaptive denoising strategies that preserve clinically meaningful morphology while selectively attenuating noise. This design not only improved robustness under diverse noise conditions but also enhanced interpretability by revealing fine-grained noise distributions within signals classified under the same quality label.

The final loss of multi-task was formulated below:

(9) $$L_{total}=\omega_{1}L_{denoising}+\omega_{2}L_{cls}+{\omega_{3}L}_{level}$$

where $\omega_{1},\;\omega_{2}\;and\;\omega_{3}\;$ are the weights of three tasks; therefore, the final loss $L_{total}$ is a weighted sum of three individual task losses, where the weights correspond to the relative importance of each task. To avoid manually tuning these weights, we adopted an uncertainty-based weighting strategy that models the homoscedastic uncertainty of each task [36]. Homoscedastic uncertainty refers to task-dependent but input-independent uncertainty, which captures the intrinsic noise level associated with each prediction task rather than variations across samples. In other words, it quantifies how inherently difficult a task is, regardless of the specific input instance. By introducing learnable task-dependent uncertainty parameters, the model automatically adjusts the contribution of each task loss during training. Tasks with higher intrinsic noise, i.e., greater uncertainty, are assigned smaller weights, preventing them from dominating the optimization process. Consequently, the final multi-task loss is formulated as follows:

(10) $$L_{total}=\sum_{i=1}^{C}\left(\frac{1}{2\sigma_{i}^{2}}L_{i}+log\sigma_{i}\right)$$

where $L_{i}$ denotes the raw loss for task *i* and $\sigma_{i}$ represents the learned uncertainty for task *i*. The logarithmic term ensures that gradients remain well-scaled during backpropagation. Through this formulation, the network learns to balance tasks automatically, eliminating the need for heuristic or manually tuned weighting coefficients.

## 4. Experiments and Results

### 4.1. Introduction of Involved Datasets

Wearable ECG Signal Quality Database (WD) and Arrhythmias Database (ArrhDB) were meticulously established by clinical cardiologists in accordance with established ECG diagnostic standards and their diagnostic experience. [37]. WD contains 300 recordings with a duration of 10 s, divided into three categories: good, medium and poor. Here, the category “Good” allows for slightly noise or artifacts, which is somewhat different from previous studies. ArrhDB was set up by screening lead I ECG signal of type ‘A’, which contains 3 major categories: sinus rhythm (category ‘N’), atrial rhythm (category ‘A’), and ventricular rhythm (category ‘V’). Paced rhythm and ventricular flutter or fibrillation (VF) were excluded from the database.

The PTB-XL dataset contains 21,837 clinical 12-lead ECG recordings of 10 s each from 18,885 patients [38]. Annotated by up to two cardiologists according to the SCP-ECG standard, it encompasses 71 diagnostic, morphological, and rhythm statements. The dataset provides official train–test splits and includes comprehensive metadata such as patient demographics and signal quality attributes.

Brno University of Technology ECG Quality Database (BUT QDB) comprises 18 long-term recordings of single-lead ECGs, collected from 15 subjects (9 females, 6 males) aged between 21 and 83 years [39]. Collected under free-living conditions using a mobile ECG device, each recording spans at least 24 h. Signal quality labels were assigned by three ECG experts, with a consensus classification into three quality categories.

PTB Diagnostic ECG Database is a collection of 549 high-resolution ECGs collected by the PTB prototype recorder, which includes 12 standard leads and 3 Frank XYZ leads [39]. The dataset covers 290 subjects (80 healthy controls) with various cardiovascular conditions. Records sampled at 1 kHz range from 38 to 115 s and are supplemented with clinical summaries.

ECG recordings from CinC2017 (PhysioNet/Computing in Cardiology Challenge 2017) were collected using the AliveCor device, which contains 8528 single-lead ECG records [40]. The available types comprised sinus rhythm, atrial fibrillation (AF), an alternative rhythm, and uninterpretable noise. ECG signals were sampled at 300 Hz, lasting from 9 s to over 60 s. Only noisy parts were used in this work.

**Figure 5:**
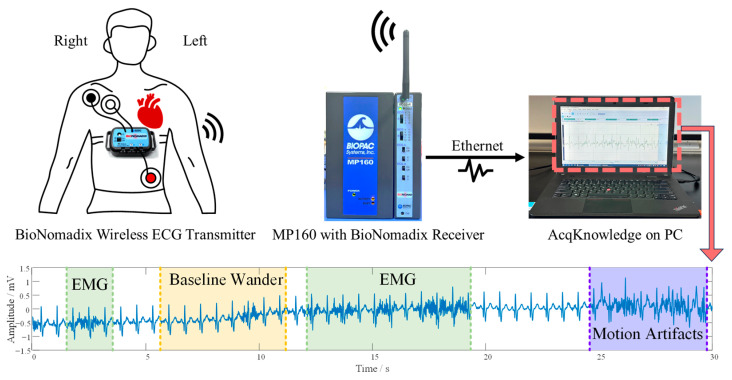
Dynamic ECG collection in wearable ECG device scenarios.

Dynamic ECG (DUT_BMELAB) was collected in our lab for a generalization test. 10 Subjects were postgraduate students, and they were asked to tighten the screw while wearing the wireless BioPac ECG device (shown in Figure 5). Sampling rate was set to 2000 Hz, and the duration of recordings was around 10 min. These ECGs were labeled as “Good”, “Medium”, and “Bad” by cardiologists from First Affiliated Hospital of Dalian Medical University. It should be noted that the rules of “Good” are more lenient, which means some acceptable levels of noise are permitted as long as they do not affect the interpretation and diagnosis.

MIT-BIH Noise Stress Test Database (NSTDB) provides three half-hour recordings of typical ambulatory ECG noise: baseline wander, muscle artifact, and electrode motion artifact [41]. Noise was captured from active volunteers using standard ECG equipment with electrodes placed to avoid visible cardiac signals.

### 4.2. Preparations for Training and Test Set

#### 4.2.1. ECG Preprocessing

When collecting ECG signals in real-world settings, it is inevitable to encounter situations such as lead detachment, which causes a long-lasting flat line issue. As a result, the first step for ECG preprocessing was the detection of a flat line. The detection algorithm used both standard deviation and amplitude thresholds to adaptively detect “flat line” conditions: if the standard deviation was below the threshold *std_thr* or the number of detected peaks was insufficient, indicating a very slow or non-existent heart rate, the algorithm proceeded to check for a “flat line” condition.

A series of filtering operations was performed. ECG signals were filtered by median filters with window sizes of 0.2 s and 0.6 s to eliminate baseline wander. A Band-pass filter with a bandwidth of 1~40 Hz for removing noises outside the main frequency band of ECG. The Savitzky–Golay filter was used for smoothing waveforms of fiducial points, and electrical isoline correction was applied. All the signals were resampled to 200 Hz to match the required input size for the model.

**Figure 6:**
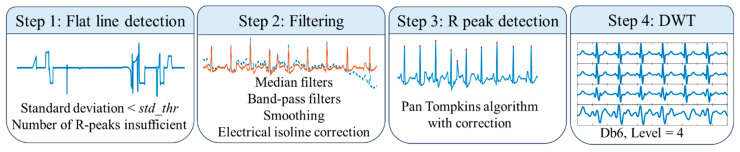
Flowchart of ECG preprocessing.

R-peak detection was implemented using the Pan–Tompkins algorithm, followed by adaptive correction. A 4-level Discrete Wavelet Transform (DWT) using the Daubechies 6 (db6) wavelet was performed for ECG delineation, as shown in Figure 6.

#### 4.2.2. Generation of Pseudo Labels

In this paper, PTB and PTB-XL were chosen to constitute the training set; therefore, the pathological information of the ECG has been fully taken into consideration to differentiate disease-induced abnormalities from noise. Official annotations for the ECG signal quality of the above-mentioned two databases were not available. Consequently, an SVM based on one-class learning was trained using ECG data from WD and ArrhDB, for selecting the clean part of PTB and PTB-XL. The 11 features used for training classifiers were derived from previous studies [42], as shown below.

- Feature 1: Kurtosis of ECG segment.
- Feature 2: Sample entropy of ECG segment.
- Feature 3: Proportion of zero crossings based on pkThr_1_ (a threshold set as 0.75* local maxima of ECG segment).
- Feature 4: Proportion of samples over pkThr_2_ (a threshold set as 0.25* local maxima of ECG segment).
- Feature 5: Proportion of zero crossings based on pkThr_2_.
- Feature 6: Proportion of signal power in 1–40 Hz to that in the entire band.
- Feature 7: Proportion of signal power in 3–20 Hz to that in 1–40 Hz.
- Feature 8: Standard deviation of ECG signal amplitude, calculated based on the R-peaks detected by the Pan and Tompkins method.
- Feature 9: Standard deviation of difference in RR intervals, calculated based on the detected R-peaks.
- Feature 10: Ratio of QRS template energy to residual sum of squares of AASeg (difference between P-wave segment and average P-wave template).
- Feature 11: Standard deviation of P-wave segment.

#### 4.2.3. Training and Testing Sets

Noisy segments from CinC2017 were included so that the model could better learn characteristics of noise and artifacts in real-world scenarios. Clean ECG signals from PTB and PTB-XL were selected, in order to add different levels of noise and attain a synthesized and balanced training set containing 3 categories of ECG segments. To be more specific, noises from CinC2017 were introduced as “Bad” category in a specific proportion in real-time during the training process of the model; BW, EM, and MA from NSTDB were also randomly added to training data online to simulate the ECG waveform under various SNR, as mentioned in Section 3.1. Consequently, the composition of training data is shown in Table 3.

### 4.3. Experiment Settings

The model architecture was illustrated in Figure 3. The input size of the single-lead ECG signal was 5 s, containing 1000 sampling points. Patch size was set to 20. The number of basic blocks in the encoder and decoder was 6 and 3, respectively. Adam optimizer was used, with a learning rate of 1×10^−3^ and weight decay of 0.05. Xavier initialization was used for parameter initialization. The network was trained for 50 epochs with a batch size of 64. For denoising loss, α is set to 2. We utilized PyTorch 2.0 (https://pytorch.org/, accessed on 14 November 2025) to implement the network.

Evaluation metrics for the signal quality assessment task and noise localization were recall, precision, F1-score, and accuracy.

(11) $$Recall=\frac{TP}{TP+FN}$$

(12) $$Precison=\frac{TP}{TP+FP}$$

(13) $$F1=\frac{2\cdot Precision\cdot Recall}{Precision+Recall}$$

(14) Acc=TP/(N+P)

Evaluation Metrics for the denoising task were percentage root mean square difference (PRD), root mean square error (RMSE), SNR of input and output, and cosine similarity (CosSim). In fact, only RMSE is a quantitative description indicator to describe how close the original and reconstructed signals are, while the other three indicators are qualitative. As a result, these three indicators possess a significant edge over generalization on cross-datasets.

(15) $$\mathrm{PRD}(x,\hat{x})=\sqrt{\sum_{i=1}^{N}{\left(x_{i}-{\hat{x}}_{i}\right)}^{2}/\sum_{i=1}^{N}x_{i}^{2}}\times 100$$

(16) $$\mathrm{RMSE}(x,\hat{x})=\sqrt{\frac{1}{N}\sum_{i=1}^{N}{\left(x_{i}-{\hat{x}}_{i}\right)}^{2}}$$

(17) $$\mathrm{SNR}(x,\hat{x})=10\mathrm{lg}\left(\sum_{i=1}^{N}x_{i}^{2}/\sum_{i=1}^{N}{\left(x_{i}-{\hat{x}}_{i}\right)}^{2}\right)$$

(18) $$\mathrm{CosSim}=-\left(\sum_{i=1}^{N}x_{i}{\hat{x}}_{i}\right)/\left(\sum_{i=1}^{N}x_{i}^{2}\sum_{i=1}^{N}{\hat{x}}_{i}^{2}\right)$$

### 4.4. Results of Signal Quality Assessment Task on Public Datasets

**Figure 7:**
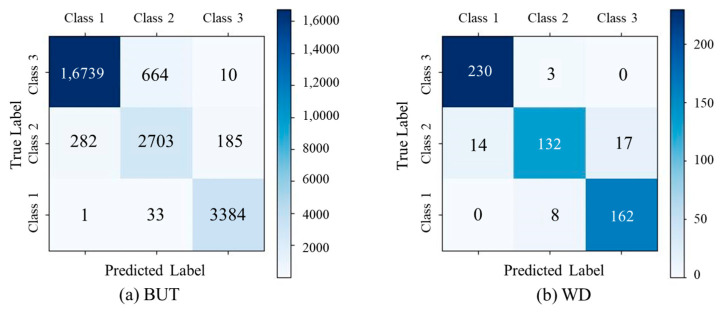
Confusion matrices of signal quality assessment task on public datasets.

The proposed method demonstrated robust and consistent performance in ECG signal quality assessment across benchmark datasets: BUT, DUT_BMELAB, and WD. On the BUT dataset (Table 4 and Figure 7a), the model achieved a weighted average F1-score of 0.9572 and an overall accuracy of 0.9568. It exhibited particularly strong performance on Class 1 and Class 3, with F1-scores of 0.9743 and 0.9775, respectively. However, Class 2 remained relatively more challenging, with a lower F1-score of 0.8295. In contrast, the WD dataset (Table 5 and Figure 7b) presented greater variability in signal quality. Despite this, the model maintained balanced performance across all classes, achieving an overall accuracy of 0.9258. Class 2 showed the most notable performance degradation, with a recall of 0.8098, highlighting its susceptibility to ambiguous or noisy signals. Across all datasets, the F1-score for Class 1 consistently exceeded 0.9644, suggesting a high degree of separability between Class 1 and the other classes. These results collectively underscored the effectiveness and generalizability of the proposed approach, while also revealing class-specific and dataset-specific challenges in real-world ECG signal quality classification.

**Figure 8:**
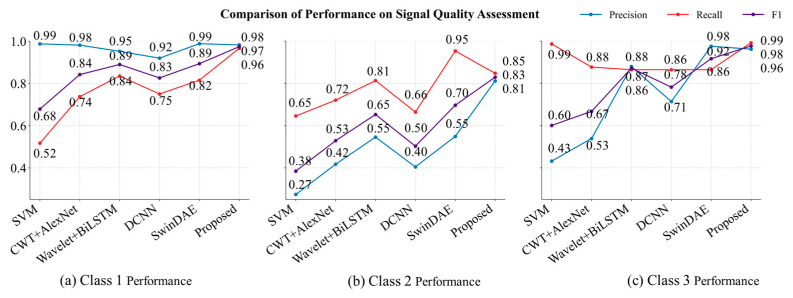
Comparison of performance on signal quality assessment.

The proposed method achieved state-of-the-art performance across all classes when compared with conventional machine learning and deep learning baselines on BUT [12,26,43,44], as illustrated in Figure 8.

For Class 1, the model attained an F1-score of 0.9743, significantly outperforming SwinDAE [12] (0.8935) and Wavelet Scattering + BiLSTM [26] (0.8898). It also achieved a near-perfect precision of 0.9825 and a recall of 0.9663, highlighting its robustness in minimizing both false positives and false negatives, and demonstrating strong reliability in detecting high-quality ECG signals.

In Class 2, which represents the most challenging category due to greater signal ambiguity, the proposed model delivered a substantial improvement with an F1-score of 0.8295, outperforming SwinDAE (0.6967, +19%) and Wavelet Scattering + BiLSTM (0.6529, +27%). Importantly, it achieved a well-balanced precision-recall tradeoff (0.8116 precision, 0.8482 recall), effectively addressing the severe imbalance observed in SwinDAE (high recall of 0.9538 but low precision of 0.5488) and the insufficient recall in DCNN [43] (0.6644).

For Class 3, the proposed approach reached near-perfect performance with an F1-score of 0.9775, surpassing SwinDAE (0.9172, +6.5%) and Wavelet Scattering + BiLSTM (0.8723, +12%). The recall reached 0.9930, with a precision of 0.9625, demonstrating the model’s exceptional sensitivity without compromising specificity. This is in contrast to SVM, which achieved a similarly high recall (0.9873) but suffered from very low precision (0.4317), leading to a much lower F1-score (0.6008). SwinDAE, although achieving high precision (0.9762), failed to match the recall level (0.8650) of the proposed method.

Overall, the proposed method consistently outperformed all baseline models in terms of F1-score across all classes, indicating superior generalization ability. It effectively addressed critical limitations of existing approaches, such as the precision-recall imbalance observed in SwinDAE for Class 2 and the inadequate class-specific adaptability of traditional models like SVM. These results underscore the effectiveness of the proposed architecture in harmonizing discriminative feature extraction with class-aware optimization.

### 4.5. Results of Denoising Task on Public Dataset

The denoising performance of the proposed method was comprehensively evaluated on the BUT dataset under SNR sampled from uniform distributions, as summarized in Table 6. The results demonstrate the model’s robustness in suppressing noise while preserving signal fidelity across a broad range of noise intensities. In high-noise conditions (−5 dB ≤ SNR ≤ 3 dB), the method achieved a substantial enhancement in output signal quality, improving the SNR from −1.95 ± 3.83 dB to 12.20 ± 2.51 dB. Although signal distortion was relatively higher in this regime—with a PRD of 25.60 ± 7.57% and RMSE of 0.07 ± 0.03 mV—the denoised signals retained essential waveform characteristics. In moderate-noise conditions (3 dB < SNR < 16 dB), the method further improved output SNR to 15.14 ± 2.12 dB, while achieving reduced distortion metrics (PRD: 18.05 ± 4.90%, RMSE: 0.05 ± 0.02 mV), indicating effective noise suppression with improved waveform preservation. For clean signals (16 dB ≤ SNR ≤ 18 dB), the model maintained high signal fidelity, yielding an output SNR of 18.40 ± 1.93 dB, with minimal distortion (PRD: 15.54 ± 3.92%, RMSE: 0.04 ± 0.01 mV), demonstrating the model’s capacity to avoid over-smoothing in low-noise scenarios.

Across all SNR conditions, the cosine similarity between original and denoised signals remained consistently high, confirming that the morphological structure of the ECG waveforms was well preserved. These findings underscored the adaptability of the proposed method to diverse noise environments, effectively balancing noise attenuation with structural integrity of the signal.

**Figure 9:**
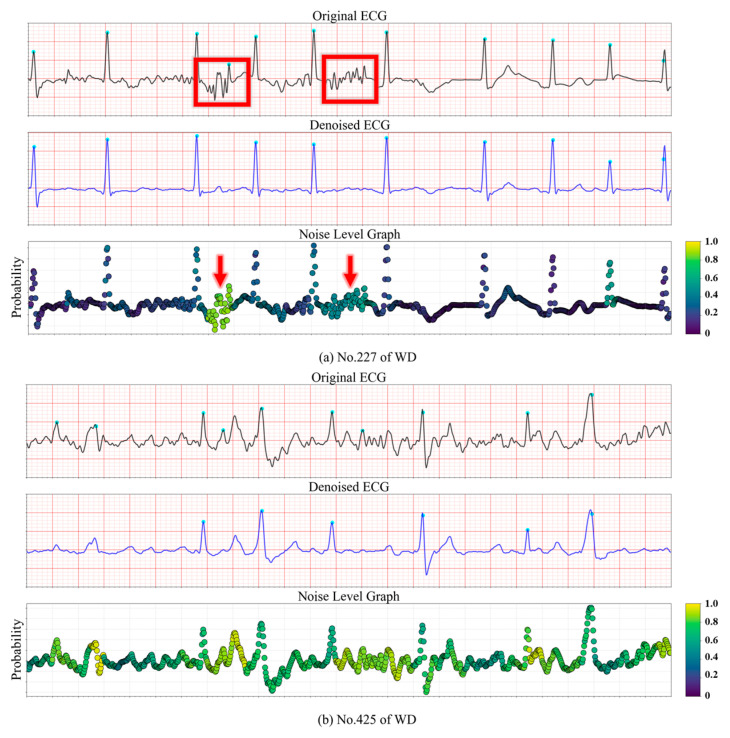

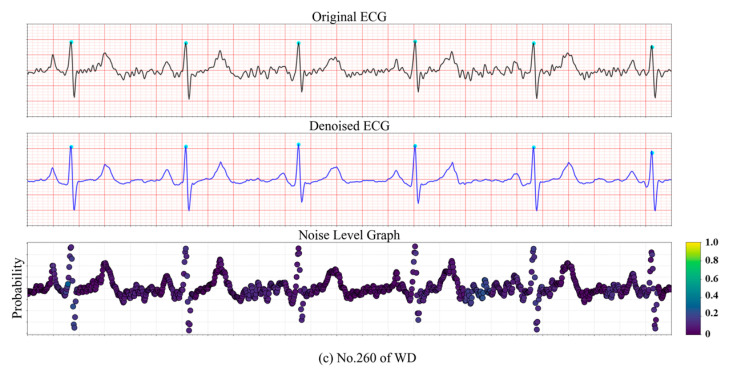
Noise level graph of a real ECG signal.

The capability of the proposed model to localize noise was further evaluated on the testing databases. Examples from the WD dataset are illustrated in Figure 9 to demonstrate the noise level estimation. In Figure 9a, the first row shows the raw ECG signal, while the second row depicts the reconstructed output ECG generated by the model. The corresponding noise level is visualized as a heatmap, where lighter colors indicate a higher probability of unacceptable noise. In Figure 9a, localized EMG interference is present, resulting in two segments being highlighted in yellow and green, indicating a high noise level. In contrast, Figure 9b contains high-frequency noise, but the model successfully identifies the signal quality as acceptable (“Good”) and effectively smooths the waveform. In Figure 9c, the majority of the ECG signal is corrupted by unknown noise sources, leading to a uniformly high estimated noise level across the entire segment.

**Figure 10:**
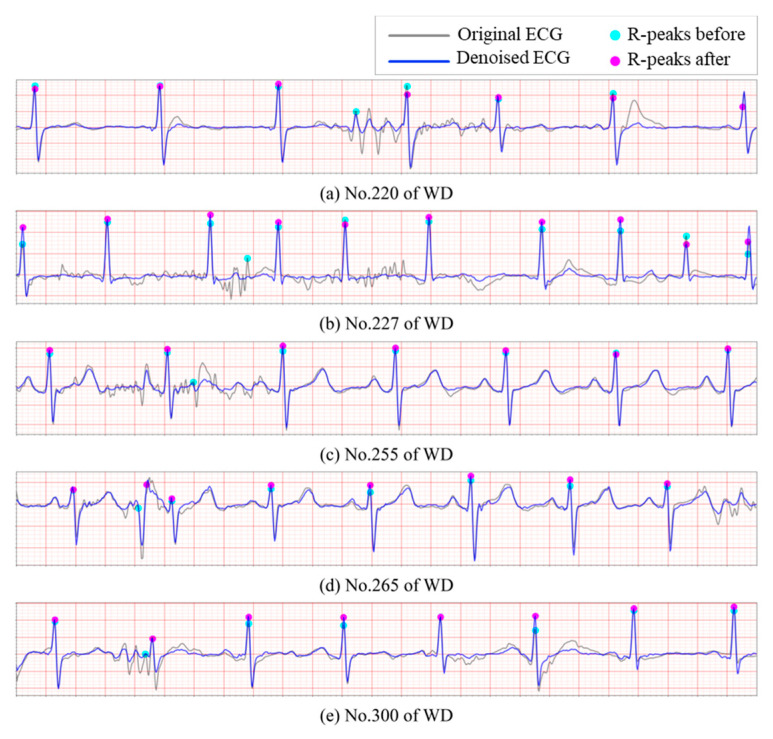
Correction of false positive R-peaks detection. (**a**–**e**): Segments of ECG signals from WD.

It is evident that the presence of noise significantly elevates the false positive rate in R-peak detection. To further evaluate the proposed model’s ability to mitigate this issue, additional experiments were conducted on WD. The Pan-Tompkins algorithm was employed to detect R peaks across the aforementioned databases, without incorporating any post-processing or correction mechanisms. As Figure 10 illustrates, noisy ECG recordings often contain sharp spikes that are erroneously identified as R peaks, leading to substantial inaccuracies in the subsequent heart rate variability (HRV) analysis. After processing through the proposed reconstruction model, a significant number of these spurious R-peak-like spikes are effectively removed, resulting in a noticeable reduction in the false positive rate. In particular, Figure 10e demonstrates a clear example where the second R peak is correctly relocated following denoising, highlighting the model’s effectiveness in preserving true cardiac events while suppressing noise-induced artifacts.

The proposed denoising method demonstrated competitive performance compared to state-of-the-art approaches, as summarized in Table 7. Our method achieved an SNR improvement of 14.13 ± 2.67 dB, surpassing traditional techniques such as the FIR filter (9.35 ± 15.46 dB) and TCDAE (13.68 ± 3.68 dB), while exhibiting significantly lower variance than MTL-NET (25.85 ± 15.49 dB). In terms of distortion metrics, the proposed method attained a PRD of 20.67 ± 6.99%, outperforming TCDAE (22.95 ± 12.8%) and substantially reducing distortion compared to DeepFilter (50.45 ± 29.60%) and CBAM-DAE (77.04 ± 36.24%). Notably, it achieved a near-optimal RMSE of 0.05 ± 0.02 mV with minimal variability, closely matching MTL-NET (0.03 ± 0.12 mV) but with greater consistency. The cosine similarity score of 0.98 ± 0.02 highlights superior waveform preservation compared to all baselines, including diffusion-based DeScoD-ECG (0.93 ± 0.09) and FIR filters of 0.88 ± 0.10. While MTL-NET shows a marginally lower PRD of 20.46 ± 34.33%, its high standard deviation suggests instability in handling diverse noise profiles. These results underscore the proposed method’s balanced trade-off between noise suppression, distortion control, and morphological fidelity, with statistically robust performance across all metrics.

### 4.6. Evaluation of Model Generalizability in Wearable ECG Scenarios

As shown in Figure 5, subjects performed specified actions during controlled ECG acquisition. This experimental design deliberately incorporated EMG interference from isometric muscle contraction and motion artifacts induced by subtle postural adjustments, thereby simulating real-world noise scenarios while ensuring measurement reproducibility. To rigorously evaluate the model’s robustness against such compound noise sources, comprehensive validation was conducted on the DUT_BMELAB dataset.

**Figure 11:**
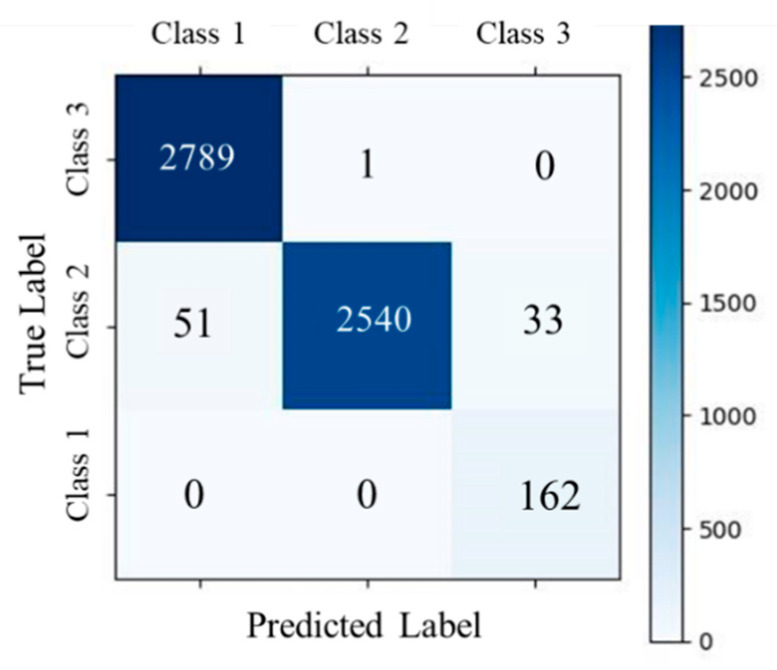
Confusion matrices of the signal quality assessment task on DUT_BMELAB.

**Figure 12:**
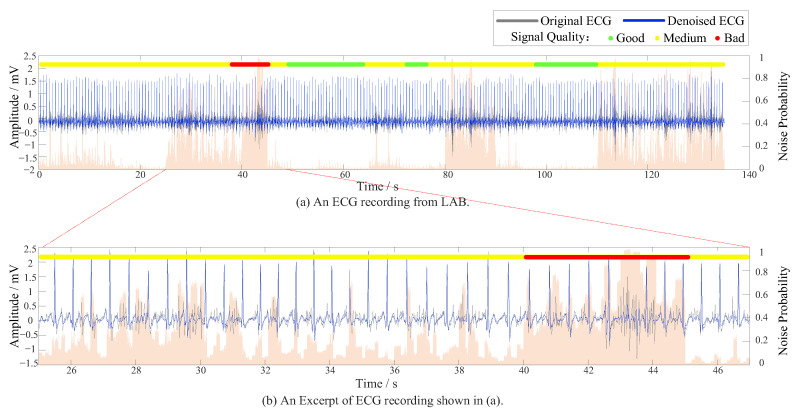
Evaluation of model generalizability in wearable ECG scenarios, where the background color represents the noise level graph.

On the DUT_BMELAB dataset (Table 8 and Figure 11), the method attained the highest overall performance, with a weighted average F1-score of 0.9849 and accuracy of 0.9848. The model achieved near-perfect recall for both Class 1 and Class 3, indicating its strong generalizability in clean and well-labeled conditions. Figure 12a visualizes a representative ECG recording from DUT_BMELAB, with Figure 12b magnifying a critical segment exhibiting EMG and motion artifacts. Background represented noise level graph and highlighted transient signal quality degradation, where orange regions correspond to physiologically unacceptable SNR. Conventional end-to-end architectures necessitate coarse-grained signal segmentation (typically 1–10 s windows), inevitably causing localized artifacts diluted by averaging. By implementing point-level noise detection through adaptive R-R interval analysis, our granular approach successfully uncovers transient noise patterns buried within individual R-R intervals, including motion-induced spikes and electrode contact losses that conventional methods overlook.

### 4.7. Model Compression via Pruning and Quantization

In real-world applications, ECG monitoring systems are often deployed on wearable or edge devices with limited computational and memory resources. Running a full-scale Transformer-based model under such conditions would introduce substantial latency and energy consumption, thereby limiting its practical usability.

To address this challenge, we applied a two-stage compression strategy consisting of structured pruning and mixed-precision quantization. Structured pruning removed redundant convolutional channels and attention heads, reducing parameters by 80%. Subsequently, INT8 quantization further decreased storage demands by converting 32-bit weights to 8-bit integers while preserving parameter count.

As presented in Table 9, the original model comprised 1.31 M parameters, requiring approximately 62.84 M multiply-accumulate operations (MACs) and achieving an average inference latency of 5.21 ms per 5 s ECG segment on a standard Intel i7 CPU. After structured pruning, parameters decreased to 0.25 million, improving latency to 3.68 ms. Further applying INT8 quantization reduced latency to 3.02 ms with negligible performance degradation. These results confirmed that the proposed compression strategy effectively reduces model complexity and storage while preserving task-specific accuracy and maintaining the integrity of the denoising, noise estimation, and signal quality assessment functions.

## 5. Discussion

This study introduced a unified multi-task learning framework that integrated ECG denoising, signal quality assessment, and fine-grained noise localization within a single model.

**Figure 13:**
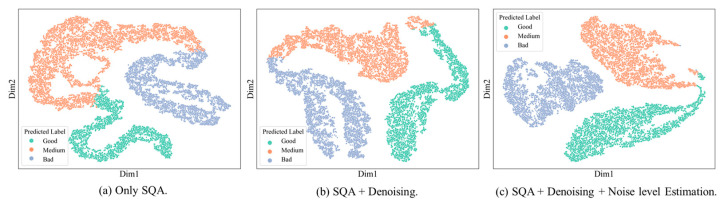
T-SNE Visualization of ECG Feature Embeddings.

1. *Robustness of Model:* from an architectural perspective, the model leveraged a hybrid design combining convolution and Transformer mechanisms to capture multi-scale features, while omitting positional encoding to reduce computational complexity. Experimental results demonstrated that the framework not only improved output SNR substantially under severe noise conditions but also preserved waveform fidelity, ensuring that diagnostic-relevant features remained intact. The advantage is most evident in Class 2 signals, traditionally the most challenging category, where the model improved F1-score by nearly 20% compared with SwinDAE, highlighting its adaptability to complex noise scenarios in clinical practice.
2. *Fine-Grained Noise Localization with Intra-Class Awareness:* the proposed noise level graph is not a superficial visualization but an interpretable intermediate representation that explicitly models the spatial uncertainty of ECG signals, offering task-relevant transparency into the model’s decision process. Specifically, it quantified the local contribution of each ECG segment to the denoising and classification objectives, thereby enabling us to interpret where and why the model identifies signal degradation or misclassification risk. A distinctive contribution of the framework lay in its intra-class awareness. Traditional quality assessment models primarily emphasized inter-class discrimination. In contrast, our model explicitly captured fine-grained variations within the same category. For example, ECGs classified as “medium quality” often exhibit heterogeneous noise sources, such as dominant EMG in one case and electrode motion artifacts in another. By coupling the denoising and noise localization tasks, the model provided segment-level insights into these intra-class differences (shown in Figure 13), enabling adaptive adjustment of denoising intensity rather than uniform processing. T-SNE visualizations of ECG feature embeddings revealed that features learned with noise localization exhibit significantly reduced intra-class dispersion compared to those without this auxiliary task. This class-aware training strategy not only improved robustness but also delivered clinically interpretable outputs that highlight subtle noise patterns within each quality category.

3. *Weak Supervision for Noise Estimation*: another important contribution lay in the dual-criterion optimization that jointly considered both SNR improvement and morphological fidelity. Unlike certain existing multi-task models that focused primarily on global error metrics, our approach achieved consistently low RMSE and near-perfect cosine similarity, ensuring that subtle waveform structures essential for clinical interpretation are preserved. This balance between quantitative accuracy and clinical interpretability underscored the practical relevance of the framework.
4. *Lightweight Design and Deployment Efficiency*: in practical deployment scenarios such as wearable or edge devices, computational and memory constraints pose significant challenges for running full-scale Transformer-based models. To address this, we implemented a two-stage compression strategy combining structured pruning and mixed-precision quantization, which reduced parameters by over 80% and latency by more than 40% without notable performance degradation. The pruned and quantized model maintained comparable accuracy while achieving a substantial reduction in computational cost and inference time, demonstrating its suitability for real-time ECG denoising and quality assessment in resource-constrained environments.

## 6. Conclusions

This work presented an interpretable deep learning framework that simultaneously performed ECG denoising, signal quality assessment, and fine-grained noise localization within a single model. By integrating weak-label-driven noise simulation, multi-task optimization, and class-aware training, the proposed method achieved superior performance. Even under high-noise conditions, the framework preserved waveform fidelity and diagnostic reliability, addressing long-standing challenges in dynamic and wearable ECG monitoring.

Through structured pruning and quantization, the model was compressed. This demonstrated its practicality for deployment in resource-constrained scenarios such as wearable devices and edge computing platforms, enabling real-time monitoring with minimal energy consumption.

In summary, this study provided not only a unified solution for ECG signal enhancement but also laid the foundation for clinically interpretable and scalable deployment in real-world settings. Future directions include refining pseudo-labeling strategies with dynamic thresholds, incorporating adaptive data selection mechanisms, and extending the framework to multi-lead ECG analysis for broader clinical applicability.

## Figures and Tables

**Table 1:** Quality and SNR criteria of ECG signals.

Category	Description	SNR (dB)
Class 1: Good	ECGs with good signal quality exhibit clear and distinct P-QRS-T waveforms, occasionally accompanied by minor noise or artifacts that do not significantly impede ECG diagnosis.	≥16
Class 2: Medium	ECGs demonstrate evident rhythmic traits, yet they are accompanied by significant signal noise, which precludes their use for morphological diagnosis.	5~14
Class 3: Bad	ECGs without clinical value.	≤−3

**Table 2:** Configuration of the model.

Stage	Layer Details	Feature Dimension	MParams
Input	(Original ECG)	1000	
Patch Embedding	[Conv1d, k = 15, s = 1] + BN + ReLU	32 × 1000	0.179
	[Conv1d, k = 7, s = 1] + BN + ReLU	64 × 1000	
	[Conv1d, k = patch_size, s = patch_size/2] + LN + ReLU	128 × 100	
Encoder	[$d_{model}$ = 128, *h* = 8, mlp_ratio = 3] × 6	100 × 128	0.992
Decoder	Linear	100 × 64	0.133
	[$d_{model}$ = 64, *h* = 8, mlp_ratio = 3] × 3	100 × 64	
Prediction head 1	Linear	100 × 20	0.003
	Unpatchify	1 × 1000	
	[Conv1d, k = 5, s = 1]	1 × 1000	
Prediction head 2	Linear	100 × 20	
	Unpatchify	1 × 1000	
Classification head	GAP + Dropout	64	
Output	(Denoised ECG)	1000	
	(Noise level Graph)	1000	
	(Signal Quality)	3	

**Table 3:** Composition of training and testing data.

	Database	Amount	Category
	PTB	4787	Good (Raw from datasets) Medium and Bad (Synthesized)
Training	PTB-XL	15,212	
	CinC2017	872	
	BUT	24,001	Good/Medium/Bad
Testing	WD	566	
	DUT_BMELAB	5576	

**Table 4:** Results of signal quality assessment on BUT (95% CI).

	Precision	Recall	F1	Acc	AUC
Class 1 (Good)	0.9825 ± 0.0041	0.9663 ± 0.0056	0.9743 ± 0.0049	0.9568 ± 0.0062	0.9873 ± 0.0032
Class 2 (Medium)	0.8116 ± 0.0107	0.8482 ± 0.0198	0.8295 ± 0.0285		
Class 3 (Bad)	0.9625 ± 0.0052	0.9930 ± 0.0024	0.9775 ± 0.0038		
Weighted Avg	0.9579 ± 0.0048	0.9568 ± 0.0046	0.9572 ± 0.0034		

**Table 5:** Results of signal quality assessment on WD (95% CI).

	Precision	Recall	F1	Acc	AUC
Class 1 (Good)	0.9426 ± 0.0061	0.9871 ± 0.0038	0.9644 ± 0.0049	0.9258 ± 0.0065	0.9779 ± 0.0035
Class 2 (Medium)	0.9231 ± 0.0093	0.8098 ± 0.0209	0.8627 ± 0.0242		
Class 3 (Bad)	0.9050 ± 0.0074	0.9529 ± 0.0059	0.9284 ± 0.0067		
Weighted Avg	0.9257 ± 0.0058	0.9258 ± 0.0054	0.9243 ± 0.0042		

**Table 6:** Results of denoising on BUT.

Noise Interval	SNR_before_ (dB)	SNR_after_ (dB)	PRD (%)	RMSE (mv)	CosSim
−5 ≤ SNR ≤ 3	−1.95 ± 3.83	12.20 ± 2.51	25.60 ± 7.57	0.07 ± 0.03	0.97 ± 0.02
3 < SNR < 16	8.89 ± 3.34	15.14 ± 2.12	18.05 ± 4.90	0.05 ± 0.02	0.98 ± 0.01
16 ≤ SNR ≤ 18	16.84 ± 0.63	18.40 ± 1.93	15.54 ± 3.92	0.04 ± 0.01	0.99 ± 0.01

**Table 7:** Comparison of denoising task (“/” indicates the absence of the corresponding metrics in the original papers.).

Method	SNR (dB)	PRD (%)	RMSE (mv)	CosSim
FIR filter	9.35 ± 15.46	45.38 ± 43.27	0.07 ± 0.09	0.88 ± 0.10
DeepFilter [45]	/	50.45 ± 29.60	/	0.90 ± 0.10
CBAM-DAE [46]	/	77.04 ± 36.24	0.06 ± 0.14	0.80 ± 0.20
DeScoD-ECG [47]	/	40.53 ± 26.26	/	0.93 ± 0.09
TCDAE [48]	13.68 ± 3.68	22.95 ± 12.8	0.06 ± 0.06	0.97 ± 0.06
MTL-NET [13]	25.85 ± 15.49	20.46 ± 34.33	0.03 ± 0.12	/
Proposed method	16.84 ± 0.63	15.54 ± 3.92	0.04 ± 0.01	0.98 ± 0.02

**Table 8:** Results of signal quality assessment on DUT_BMELAB (95% CI).

	Precision	Recall	F1	Acc	AUC
Class 1 (Good)	0.9820 ± 0.0035	0.9996 ± 0.0005	0.9908 ± 0.0021	0.9848 ± 0.0037	0.9891 ± 0.0026
Class 2 (Medium)	0.9996 ± 0.0008	0.9680 ± 0.0049	0.9835 ± 0.0036		
Class 3 (Bad)	0.8308 ± 0.0098	0.9908 ± 0.0011	0.9076 ± 0.0075		
Weighted Avg	0.9859 ± 0.0029	0.9848 ± 0.0027	0.9849 ± 0.0025		

**Table 9:** Original vs. compressed model configuration.

Model Variant	Params (M)	Storage (MB)	MACs (M)	Latency_CPU (ms)	Accuracy (%)	SNR (dB)
Original	1.31	4.99	62.84	5.21	95.68	16.84
Pruned	0.25	0.99	27.06	3.68	93.75	15.73
Pruned + INT8	0.25	0.25	27.06	3.02	93.11	15.35

## Data Availability

Our code and DUT_BMELAB Database will be available on request.
